# Fragment detection of Coleopteran and Triatomine insects in experimentally contaminated acai pulp and sugarcane juice

**DOI:** 10.1590/0037-8682-0119-2019

**Published:** 2019-12-20

**Authors:** Elaine Cristina de Mattos, Maria Aparecida Moraes Marciano, Vilma dos Santos Menezes Gaiotto Daros, Cristiane Castro Faccini, Angela Maria Lourenço, Vera Lucia Pereira-Chioccola

**Affiliations:** 1 Instituto Adolfo Lutz, Centro de Laboratório Regional de Santo André, Laboratório de Microscopia Alimentar, Santo André, SP, Brasil.; 2 Instituto Adolfo Lutz, Núcleo de Morfologia e Microscopia, São Paulo, SP, Brasil.; 3 Instituto Dante Pazzanese de Cardiologia, Laboratório de Doença de Chagas Elias Boainain, São Paulo, SP, Brasil.; 4 Instituto Adolfo Lutz, Centro de Parasitologia e Micologia, São Paulo, SP, Brasil.

**Keywords:** Coleoptera, Euterpe, Triatominae, Polymerase Chain Reaction, Food, Foodborne Diseases

## Abstract

**INTRODUCTION:**

Oral transmission of acute Chagas disease is an emerging public health concern. This study aimed to detect insect fragments in experimentally contaminated food, by comparing triatomines with other insects.

**METHODS:**

Food samples were experimentally contaminated with insects, processed to recover their fragments by light filth, and analyzed by microscopy and Polymerase Chain Reaction (PCR).

**RESULTS:**

Morphological differences between coleopteran and triatomine insects were observed in microscopic images. PCR was efficient in amplifying Triatominae DNA in the experimentally contaminated food.

**CONCLUSIONS:**

This methodology could be utilized by food analysts to identify possible insect contamination in food samples.

The occurrence of insects in food inspection indicates problems in hygienic-sanitary conditions and other potential food safety problems^1^. A particularly severe food safety concern is the oral transmission of acute Chagas disease by ingestion of contaminated food. Although the vectorial transmission of *Trypanosoma cruzi* is traditionally the best known, the oral route has been an important mode of transmission in Brazil recently^2^. Typically, foods are contaminated with *T. cruzi* during harvesting, storage, transport, or preparation^3^. The detection of insect fragments in samples of acai pulp, sugarcane juice, and other foods is considered an indicator of poor hygienic-sanitary conditions and an effective method for investigating outbreaks of acute Chagas disease. Additionally, molecular methods have contributed to the detection and quantification of pathogens in foods, as demonstrated in a previous study detecting *T. cruzi* in acai pulp and sugarcane juice^4^.

These data allowed us to perform an experimental contamination for subsequent detection of insect fragments. The purpose was to investigate whether microscopic and molecular methods could detect the presence of insect fragments in foods. The investigations were focused on triatomine insects. Furthermore, these experiments were compared with those involving coleopteran insects, which are typically found in stored grains and other foods.

For the experiments, aliquots (500 g) of acai pulp or sugarcane juice (500 mL) were crushed in a blender for 2 min, with two-third instar nymphs of *Triatoma infestans* (Hemiptera, Reduviidae) from the Laboratório de Doença de Chagas Elias Boainain, Instituto Dante Pazzanese de Cardiologia, Sao Paulo, Brazil. In parallel, two other samples of acai pulp (500 g) and sugarcane juice (500 mL) were crushed with a mixture of Coleoptera insects containing two adult *Sitophilus* spp., two adult *Tribolium* spp., and four adult *Trogoderma granarium*. These insects were derived from previous analyses performed at Centro de Laboratório Regional de Santo André, Instituto Adolfo Lutz, Sao Paulo, Brazil. To isolate light filth, insect fragments in acai pulp samples were recovered as described previously^5^. The insect fragments in sugarcane juice were recovered using the methodology 16.5.18 (method 972.35) described previously^6^, with modifications.

Polymerase chain reaction (PCR) was performed using aliquots of 500 µL from each mixture. The methodology for DNA extracted from the contaminated foods was performed as described previously^4^. Triatominae DNA was identified using the primer set that included forward Reduv 18S - (5´AAATTACCCACTCCCGGCA3´) and reverse Reduv 18S (5´TGGTGUGGTTTCCCGTGTT3´), which resulted in an amplification of 883 bp^7^. Amplifications were performed in a final volume of 25 μL containing 0.25 μL of each primer, 5 μL of each DNA template, and 12.5 μL of Gotaq®green Master Mix (Promega®), each containing Taq DNA polymerase in Tris-HCl 10 mM, pH 8.5; KCl 50 mM; MgCl_2_ 1.5 mM; and dATP, dGTP, dCTP, dTTP 200 mM. Reactions were performed in a Veriti® Thermal Cycler (Applied Biosystems®). DNA extracted from acai pulp or sugarcane juice (approximately 100 ng/μL) was tested in duplicate. The cycling conditions were as follows: 94 °C for 5 min, followed by 35 cycles of 94 °C for 30 s, 47.5 °C for 30 s, 72 °C for 30 s, and a final cycle of 72 °C for 7 min. Next, PCR products were electrophoresed in 2% agarose gels in Tris-Borate-EDTA buffer and stained with ethidium bromide. The fragment size was based on a comparison with a 100-bp ladder. 

The PCR controls included two positive controls (DNA extracted directly from *T. infestans* and *Rhodnius neglectus*) and three negative controls (DNA extracted from *Lasioderma serricorne*, *Trogoderma granarium,* and a food sample without insect fragments). In each PCR run, a blank control consisted of DNA-free water and a PCR mix. Separate rooms were used for i: DNA extraction; ii: PCR mix and primer preparation; iii: adding DNA from samples and positive control (DNA extracted from insects); and iv: post-PCR agarose gel electrophoresis analysis. DNA samples were assayed in duplicate at least twice to determine reproducibility. 

The food samples were experimentally contaminated with insects and subsequently investigated for light filth. This methodology allowed for fragments of triatomines to be distinguished from coleopteran ones. The stereoscopy (Figures 1A and 1B) and light microscopy (Figures 1C, 1D, 1E, and 1F) images showed the distinction between the triatomines integument and elytra (fused wings) of coleopteran insects. The elytra of *Sitophilus* spp. were covered uniformly (bristle inserts). Meanwhile, the *T. infestans* integument was composed of many folds, with slight inclinations and randomly distributed points. Fragments of triatomines were flexible at the touch of a needle and appeared opaque and grayish (Figures 1A, 1C, and 1D). Fragments of coleoptera were firm, stable in shape, and with shiny and brown surface (Figures 1B, 1E, and 1F). 

**FIGURE 1:**
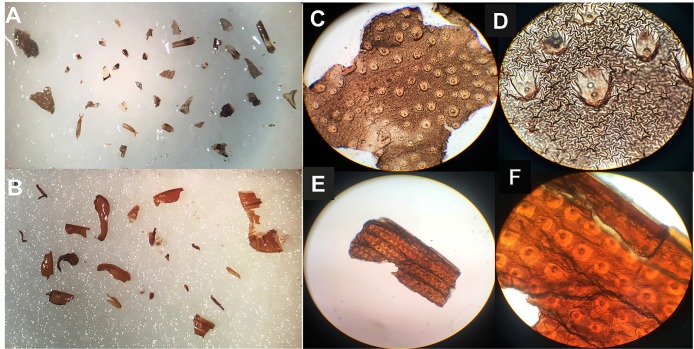
Optical stereoscopy **(A and B)** and light microscopy **(C to F)** images of insect fragments recovered from foods. Integument of *T. infestans* **(Panels C and D)**; and Coleoptera elytra **(Panels E and F)**. Magnification: **(A and B)** 15; **(C and E)** 100; **(F and D)** 400-fold.

The trichogen cells, i.e., the epidermal inclusions responsible for tactile sense and hearing, were distributed throughout the body of both coleopteran and triatomine insects. The structures were morphologically different between coleoptera (Figures 2 A and 2B) and triatomine (Figure 2C). 

**FIGURE 2:**
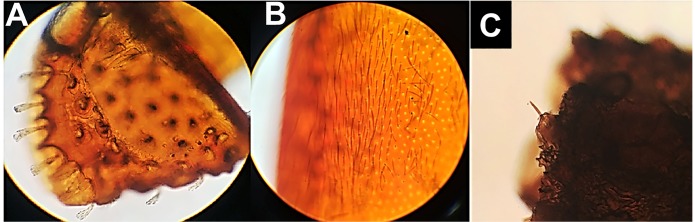
Light microscopy images of insects recovered from foods. Trichogenic cells (hair) in elytra fragments of *Sitophilus* spp. **(Panel A)**; *Tribolium* spp. **(B)**; and *T. infestans* **(C)**. Magnification: **(A, B and C)** 400-fold.

The light filth methodology could not identify insect genus or species by microscopy, as the fragments recovered were small. However, the integument fragments of triatomine insects differed from those in coleoptera and other crop pests, such beetles and Lepidoptera.

Insect genus was investigated by PCR. Figure 3 shows the amplified products of an 883-bp target region of the Reduviidae 18S in acai pulp and sugarcane juice contaminated with *T. infestans.* Amplified products were shown in positive controls of DNA from *T. infestans* and *R. neglectus*. The DNA extracts of foods infected with Coleoptera insects and that of negative controls (*L. serricorne* and *T. granarium*) were negative. 

**FIGURE 3:**
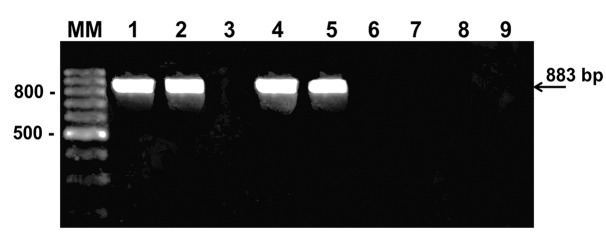
Amplified PCR products of an 883-bp target region of Reduviidae 18S (Reduv-18S). Acai pulp (line 4) and sugar cane juice (Line 5) contaminated with *Triatoma infestans.* Positive controls: *Triatoma infestans* (line 1) and *Rhodnius neglectus* (line 2). Negative controls: blank control (line 3), *Lasioderma serricorne* (line 6), *Trogoderma granarium* (line 7), acai pulp (line 8), and sugar cane juice (Line 9) contaminated with Coleoptera insects (mixture). DNA fragments were resolved in 2% agarose gels stained with ethidium bromide. Lane MM, 100-bp ladder.

Although laws regarding food quality and safety standards for food production have been implemented in Brazil, especially for fruit juices and pulps, a considerable amount of acai pulp consumed in Northern region of Brazil does not undergo pasteurization. This compromises the quality guarantee of the product and is associated with outbreaks of orally transmitted acute Chagas disease^8^ ^,^ ^9^. Thus, studies that evaluate pests and vector contamination in foods contribute significantly to the improvement of plant-based foods and beverages, especially when these products may serve as vehicles for the oral transmission of Chagas disease.

According to “Good Manufacturing Practices” in some food products, the presence of insect fragments indicates failures that may occur at various stages of the production chain. Pieces of elytra, thorax, jaws, legs, antennae, cephalic capsules, and rarely, whole insects have been observed^10^.

The process of identifying insect species recovered from food involves microscopic comparison of insect fragments with reference insect specimens. Precise identification of insects is important for regulatory purposes, i.e., for assessing the etiology of food contamination and establishing the degree of potential risk to consumers^1^. 

The identification of insects in products by fragments, species identification, and cuticle characterization is based on the absence or presence of cell structures as translucency, bristles, bristles bases, spines, and patterns of the integument surface. Insect elytra are considered important for contamination diagnosis, as they contribute to the identification of species from which they originated. Analysis is improved when one determines the stage of production at which the food was contaminated^11^ ^,^ ^12^.

Considering the time of analysis and the substantial analytical experience necessary for the identification of insect fragments in foods, Park et al. (2016)^1^ developed an algorithm for identifying coleopteran species using microscopic images of elytra fragments, as these structures constitute a large portion of the insect's body and comprise characteristic patterns. Moreover, elytra fragments are more typically recovered from processed food products than from other parts of the body owing to their hardness.

As this study aimed to investigate triatomine insects in food samples involved in outbreaks related to acute Chagas disease caused by oral transmission, acai pulp and sugarcane juice samples were experimentally contaminated with *T. infestans*, as this species is well-adapted in laboratorial conditions. The experiments were compared with coleopteran insects owing to the high contamination index of these insects in food from fields and in storage.

Microscopic analyses could characterize the differences between the *T. infestans* integument and coleoptera elytra. Other differences included hair in the epidermis, and the texture, color, hardness, and size of insect fragments. 

The color pattern of the insect species is important for identification. In triatomines of certain species, the color of the first pair of wings may aid in identification. However, *T. infestans* presents three chromatic patterns: mataral, clair, and darkmorph^13^. These fragments are similar as those in the species of the Triatominae subfamily. In addition to the coloration pattern, cuticular extensions of insects, such as hair, have been studied and classifications have been proposed for use as an identification tool, including for taxonomy. Despite several morphological studies in triatomine insects, knowledge regarding the distribution and morphological types of these structures in the subfamilies remains scarce and fragmented^7^ ^,^ ^14^.

Insect fragments in food are analyzed according to methods recommended by the AOAC (2016)^6^ ^,^ ^8^. However, these techniques allow for the recovery of fragments that are not typically intact, rendering it difficult to identify the insects to which these structures belong. 

The quantity and description of isolated insect fragment has been reported^8^ ^,^ ^10^ ^,^ ^11^; however, insect fragments present in food products have not been morphologically described.

The use of molecular methods for the identification of insect fragments in food products is not frequent. Balasubramanian et al. (2007)^15^ contaminated wheat flour samples with different concentrations of two Coleoptera species and concluded that PCR was efficient in detecting both species only at the highest concentration tested.

These results demonstrate the possibility of morphologically characterizing triatomine fragments in food products that are experimentally contaminated with insects. In addition, the PCR results indicated a good method for diagnostic complementation. This methodology for the specific identification of triatomine fragments allows for food analysts to identify possible contamination by insect vectors in food samples, as it is important to investigate indirect causal relationships of etiological foodborne transmission agents during disease outbreaks in humans. Additionally, the control of food hygienic conditions will be improved in regions where the methodologies that ensure health safety are infeasible from an economic perspective.
